# Comparison of Visual Performance of Multifocal Intraocular Lenses with Same Material Monofocal Intraocular Lenses

**DOI:** 10.1371/journal.pone.0068236

**Published:** 2013-06-28

**Authors:** Tomofusa Yamauchi, Hitoshi Tabuchi, Kosuke Takase, Hideharu Ohsugi, Zaigen Ohara, Yoshiaki Kiuchi

**Affiliations:** 1 Department of Ophthalmology, Tsukazaki Hospital, Himeji, Japan; 2 Department of Ophthalmology, Hiroshima University, Hiroshima, Japan; Zhongshan Ophthalmic Center, China

## Abstract

**Purpose:**

To compare the visual performance of multifocal intraocular lenses (IOLs) and monofocal IOLs made of the same material.

**Methods:**

The subjects included patients implanted with either Tecnis® monofocal IOLs (ZA9003 or ZCB00) or Tecnis® multifocal IOLs (ZMA00 or ZMB00) bilaterally. We conducted a retrospective study comparing the two types of IOLs. The multifocal group included 46 patients who were implanted with Tecnis® multifocal IOLs bilaterally. The monofocal group was an age- and sex-matched control group, and included 85 patients who were implanted with Tecnis® monofocal IOLs bilaterally. Lens opacity grading, the radius of corneal curvature, corneal astigmatism, axial length and the refractive status were measured preoperatively. Pupil size, ocular aberrometry, distance, intermediate and near visual acuity, contrast sensitivity with and without glare and the responses to a quality-of-vision questionnaire were evaluated pre- and postoperatively.

**Results:**

The uncorrected near visual acuity was significantly better in the multifocal group, whereas both the corrected intermediate and near visual acuity were better in the monofocal group. Contrast sensitivity (with and without glare) was significantly better in the monofocal group. The rate of spectacle dependency was significantly lower in the multifocal group. There were no significant differences between the two groups regarding most items of the postoperative quality-of-vision questionnaire (VFQ-25), with the exception that the patients in the monofocal group reported fewer problems with nighttime driving.

**Conclusions:**

The multifocal IOLs used in this study reduced spectacle dependency more so than monofocal IOLs and did not compromise the subjective visual function, with the exception of nighttime driving.

## Introduction

Currently, the goal of cataract surgery is to provide fast and complete visual rehabilitation without surgical complications with minimal postoperative refractive errors. Several materials are used to compensate for the loss of accommodation from implantation of an intraocular lens (IOL), including multifocal IOLs [1]–[6], accommodating IOLs [7] and monovision [8], [9]. Among these approaches, bilateral implantation of multifocal IOLs is the most popular. Multifocal IOLs generate different foci to address the visual limitation at near and intermediate distances that occurs with classic monofocal IOLs. Indeed, multifocal IOLs have been shown to provide good distance and near functional vision without the use of corrective lenses [5], [6], [10]–[15]. However, some optical side effects have been reported, including decreased contrast sensitivity, glare disability and halos [16]–[20], which can significantly affect the patient’s visual performance and satisfaction. The aim of this study was to evaluate the benefits and disadvantages of multifocal IOLs compared with monofocal IOLs.

Tecnis® monofocal IOLs (ZA9000 series, ZCB00) are foldable three-piece (ZA9000 series) or one-piece (ZCB00) acrylic IOLs with 6.0-mm optics. They have an aspheric, modified prolate anterior surface that is designed to minimize spherical aberrations and improve contrast sensitivity after cataract surgery [21]. In contrast, Tecnis® multifocal IOLs (ZMB00, ZMA00) are foldable three-piece (ZMB00) or one-piece (ZMA00) diffractive multifocal acrylic IOLs with 6.0-mm optics and an aspheric, modified prolate anterior surface. Except for their additional bifocal diffraction gratings, these multifocal lenses are identical to Tecnis® monofocal IOLs with respect to materials and optical design. In this study, we compared the visual function and vision-related quality of life in patients receiving these two types of IOLs.

## Methods

### Patients and Methods

We designed a case-controlled study to include the eyes of bilateral cataract patients who requested a surgical refractive solution to improve near and far vision. The patients underwent cataract surgery at the Cataract Unit of the Department of Ophthalmology, Tsukazaki Hospital between April 2009 and January 2012. The exclusion criteria included ocular disease that affected the visual function and astigmatism greater than 1.50 diopters.

### Preoperative Examination

Preoperatively, all patients received a full ophthalmologic examination, including evaluations of the radius of corneal curvature, corneal astigmatism, axial length, refractive status, pupil size, ocular aberrometry, distance, intermediate and near visual acuity and contrast sensitivity with and without glare disability, an anterior segment evaluation using a slit lamp, tonometry and indirect fundoscopy. Lens opacity was graded using the Lens Opacities Classification System III (LOCS III) [22], and the average of the right eye and left eye was used for each patient. For the radius of corneal curvature, corneal astigmatism, axial length, refractive status, visual acuity and contrast sensitivity, we measured these parameters twice within two months before cataract surgery, then took the mean of these two measurements and used the average of the right eye and left eye means. The quality of vision was evaluated using the Japanese version of the 25-item National Eye Institute Visual Function Questionnaire (NEI VFQ-25) [23]. The NEI VFQ-25 was administered by experienced technicians or nurses in a face-to-face setting.

Uncorrected distance visual acuity (UDVA) and corrected distance visual acuity (CDVA) were measured at 5.0 m. Uncorrected intermediate visual acuity (UIVA) and corrected intermediate visual acuity (CIVA) were measured at 0.5 m. Uncorrected near visual acuity (UNVA) and corrected near visual acuity (CNVA) were measured at 0.3 m. Visual acuity was measured using the decimal visual acuity chart, and the measured decimal values were converted to the logarithm of the minimum angle of resolution (logMAR) scale. The radius of corneal curvature, corneal astigmatism and refractive status were measured using a KR-8900 autorefractor keratometer (Topcon, Tokyo, Japan). Axial length was measured using IOL Master (Carl Zeiss, Oberkochen, Germany) and AL-3000 biometers (TOMEY, Nagoya, Japan). Contrast sensitivity (with and without glare) was measured using a CGT-1000 contrast glare tester (Takagi Seiko, Nakano, Japan), and pupil size and ocular aberrometry were measured using a KR-1W wave-front analyzer (Topcon, Tokyo, Japan). All measurements were obtained by experienced technicians.

### IOLs and Surgical Technique

The patients chose to be implanted with either monofocal or multifocal IOLs after they had been informed of the advantages and disadvantages associated with each type. The monofocal group was an age- and sex-matched control for the multifocal group, and the patients in this group received Tecnis® monofocal IOLs (ZA9003 or ZCB00) bilaterally, while the multifocal group received Tecnis® multifocal IOLs (ZMA00 or ZMB00) bilaterally. We did not discriminate between three-piece IOLs (ZA9003 and ZMA00) and one-piece IOLs (ZCB00 and ZMB00). The cataract surgeries were performed by six experienced surgeons. The surgical procedures consisted of topical anesthesia, the creation of a scleral or corneal incision of 2.8 to 3.2 mm, 5.0-mm continuous capsulorhexis, phacoemulsification cataract extraction and IOL implantation with an injector.

### Postoperative Examination

With respect to visual acuity (distance, intermediate and near vision), contrast sensitivity and glare disability, we measured the parameters twice (at postoperative weeks 10 and 14), calculated the average values and then used the average of both eyes. Pupil size and ocular aberrometry were measured at 14 weeks, and the average of both eyes was used. The NEI VFQ-25 was evaluated at 18 weeks. Spectacle use was evaluated at 18 weeks by inquiring how often the patient used spectacles for distance, intermediate and near vision (with possible responses of ‘never,’ ‘occasionally’ or ‘always’).

### Statistical Analyses

The statistical analyses were performed using a commercially available software program (JMP, version 10.0; SAS Institute Inc., USA).

### Ethics Statement

The procedures used in this study conformed to the tenets of the Declaration of Helsinki and were approved by the Ethics Committee of Tsukazaki Hospital. Signed informed consent was obtained from all subjects after they were informed of the procedures and possible side effects. This study is registered as UMIN000006853: “Comparison between multi and single focal intraocular lens implantation.”

## Results

Forty-six subjects were implanted with multifocal IOLs (ZMA00∶32 patients; ZMB00∶14 patients) bilaterally and 85 subjects were implanted with monofocal IOLs (ZA9003∶47 patients; ZCB00 38 patients) bilaterally. The mean ages of the respective groups were 67.46±7.56 and 67.84±5.89 years. There was no significant difference between two groups (P = 0.7442; Student’s *t*-test). The percentage of female patients was 73.91% (34/46) in the multifocal group and 74.12% (63/85) in the monofocal group. There were no significant differences between the two groups in terms of the patients’ age or sex (P = 0.7442 by Student’s *t-*test and *p* = 0.9797 by the chi-square test, respectively).

Regarding the measurements of the preoperative radius of corneal curvature, corneal astigmatism, axial length, spherical equivalent, pupil size, ocular aberrometry, visual acuity (distance, intermediate and near vision), contrast sensitivity, glare disability and LOCS III score, no significant differences were observed between the two groups after correction for age and sex using multiple regression analyses. The NEI VFQ-25 scores were not significantly different between the two groups for any question following the same correction procedure. Surgeries were performed through corneal incisions in 44 of the 46 patients in the multifocal group, and in 80 of the 85 patients in the monofocal group. Surgeries through scleral incisions were performed in two multifocal patients and five monofocal patients. There were no significant differences in the corneal/scleral incision ratio between the two groups (chi-square test).

**Figure 1:**
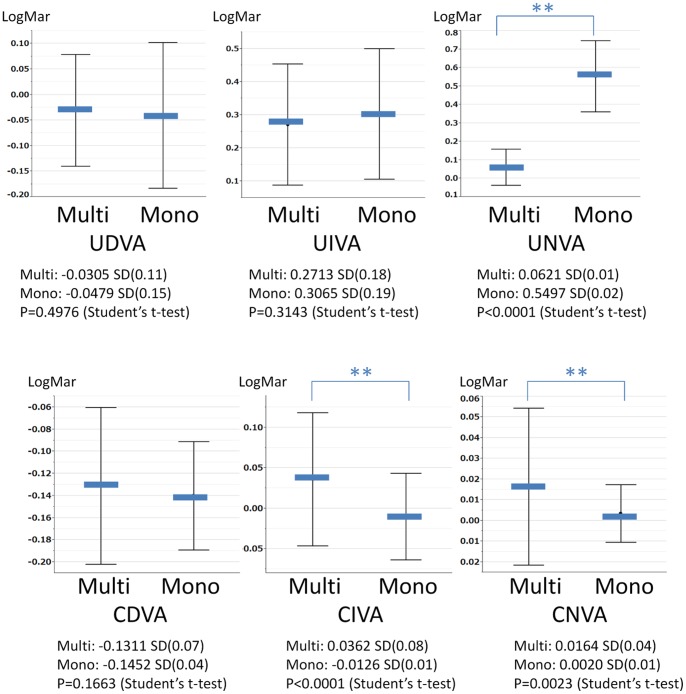
Uncorrected and corrected visual acuity of the multifocal and monofocal groups (mean±SD).

**Figure 2:**
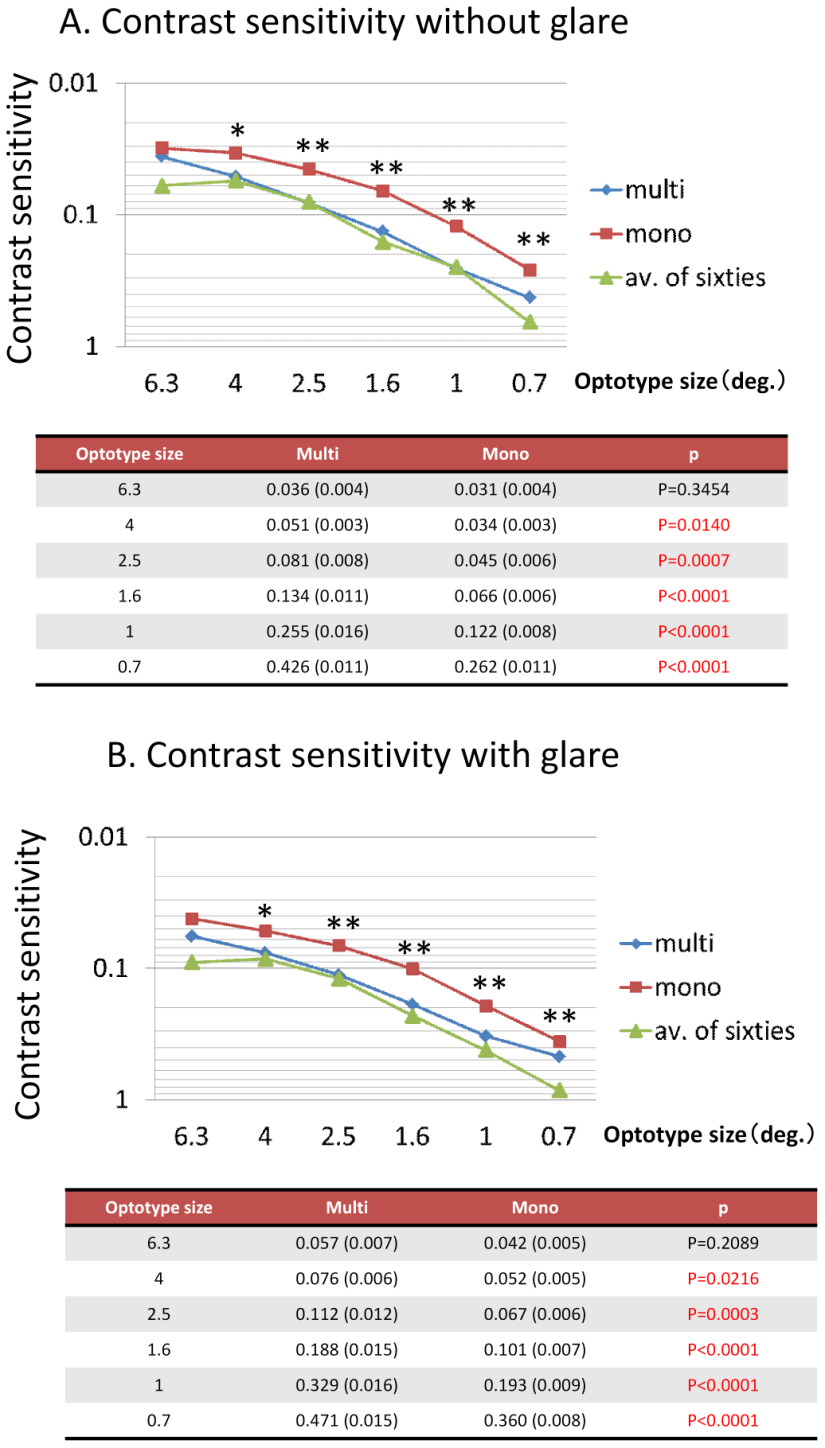
Postoperative contrast sensitivity with and without glare. The average value for 60-year-old normal subjects is cited from a report by Takahashi [29].

Figure 1 shows the results of a comparative analysis of postoperative visual acuity between the groups. There were no statistically significant differences in UDVA, UIVA or CDVA. UNVA was significantly better in the multifocal group, whereas CIVA and CNVA were better in the monofocal group. Figure 2 shows the findings of postoperative contrast sensitivity with and without glare. Contrast sensitivity was significantly better in the monofocal group than in the multifocal group (both with and without glare). Table 1 shows the rate of spectacle use in both groups. Spectacle dependency was defined as the percentage of patients who “always” or “occasionally” use eyeglasses. The rate of intermediate and near spectacle dependency was significantly lower in the multifocal group. Distance spectacle dependency was also lower in the multifocal group; however, the magnitude of this difference was not significant. Tables 2 and 3 show the postoperative scores of the NEI VFQ-25. The subjects who were evaluated for their driving scores were patients who drove daily (30 patients in the multifocal group and 49 patients in the monofocal group). There were no significant differences in any subscale between the groups, and for only one question related to nighttime driving did the monofocal group significantly outperform the multifocal group. Regarding pupil size and ocular aberrometry, there were no significant differences between the two groups.

**Table 1:** Spectacle dependency in both groups.

		Never (N)	Occasionally (N)	Always (N)	Spectacle dependency	*p*-value
Distance	Multi	42	2	2	8.7%	
	Mono	67	4	14	21.2%	0.068
Intermediate	Multi	45	0	1	2.2%	
	Mono	68	8	9	20.0%	0.005
Near	Multi	39	6	1	15.2%	
	Mono	10	10	65	88.2%	<0.0001

The *p*-values were determined using the chi-square test.

**Table 2:** Postoperative scores of the NEI VFQ-25.

	Multi	Mono	*p*-value
**Composite**	91.3 (0.9)	91.1 (0.7)	*p* = 0.8180
**General health**	63.3 (2.0)	59.8 (1.4)	*p* = 0.1472
**General vision**	80.4 (1.8)	79.1 (1.1)	*p* = 0.4828
**Ocular pain**	90.9 (1.6)	89.5 (1.4)	*p* = 0.5212
**Near vision**	93.2 (1.6)	94.2 (1.0)	*p* = 0.5858
**Distance vision**	81.7 (2.7)	78.2 (0.7)	*p* = 0.2776
**Social function**	93.4 (2.1)	90.1 (1.6)	*p* = 0.2183
**Mental health**	95.2 (1.3)	96.3 (0.8)	*p* = 0.4757
**Role limitation**	96.9 (1.1)	96.5 (1.0)	*p* = 0.7699
**Dependency**	99.1 (0.6)	98.7 (1.0)	*p* = 0.7848
**Driving (n = 79)**	88.3 (2.5)	92.9 (1.4)	*p* = 0.0831
**Color vision**	98.4 (0.9)	99.4 (0.4)	*p* = 0.2437
**Peripheral vision**	95.7 (1.4)	94.1 (1.7)	*p* = 0.5476

The values are presented as the means (SE) of n = 131 (46 patients in the multifocal group; 85 patients in the monofocal group), except where otherwise noted. The *p*-values were determined using Student’s *t*-test.

**Table 3:** Detailed summary of driving scores according to general, daytime, nighttime and adverse conditions (e.g., rain or traffic jams).

	Multi	Mono	*p*-value
**Driving, general**	88.3 (2.5)	92.9 (1.4)	*p* = 0.0831
**Daytime**	95.8 (1.7)	97.9 (1.0)	*p* = 0.2682
**Nighttime**	79.5 (4.1)	90.9 (2.2)	***p*** ** = 0.0086**
**Adverse conditions**	89.4 (2.8)	92.4 (2.0)	*p* = 0.3698

The values are presented as the means (SE). The *p*-values were determined using Student’s *t*-test. The subjects evaluated for their driving scores were limited to those who drove daily (n = 79; 30 patients in the multifocal group; 49 patients in the monofocal group).

## Discussion

In this study, we evaluated the clinical outcomes of two IOLs, Tecnis® multifocal IOLs and Tecnis® monofocal IOLs, both of which consist of the same material. UNVA was better in the multifocal group, as expected. On the other hand, CIVA and CNVA were worse in the multifocal group. There are many reports comparing corrected visual acuity between patients with multifocal IOLs and those with monofocal IOLs. Most recent studies using modern multifocal IOLs have reported that the CNVA of multifocal groups is not inferior to that of monofocal groups [24], [25]. Our results are apparently inconsistent with this finding; however, we consider it reasonable that the corrected visual acuity achieved with multifocal IOLs is worse than that achieved with monofocal IOLs. Diffractive multifocal IOLs divide light into two foci. Tecnis® multifocal IOLs use 41% of incoming light for distance vision and 41% for near vision, while 18% is lost. Therefore, compromising optical performance is unavoidable to some extent. The CDVA and CNVA values obtained in the present study were better than those reported in past studies [24], [25] in both the multifocal and monofocal groups. This may explain why differences were more easily detected.

The CGT1000 is an automatic contrast sensitivity measuring instrument. It displays a circular optotype that consists of three colored concentric circles that differ in brightness. This instrument changes the size of the optotype and the brightness contrast, after which patients are asked whether they can distinguish any differences in brightness. The device can measure contrast sensitivity at six sizes and 13 contrast levels with or without glare [26]. In this study, the contrast sensitivity measured with the CGT1000 was worse in the multifocal group at most frequencies both with and without glare, a finding that is consistent with previous reports [27], [28]. Packer et al. compared the contrast sensitivity between subjects implanted with either Tecnis® multifocal ZM900 IOLs or CeeOn 911A monofocal IOLs, which share the same optic material and haptic and edge design [28] (ZM900 is a silicone IOL that has the same optical design as the multifocal IOL used in the present study.). The authors reported that the mean contrast sensitivity was worse in the ZM900 implanted subjects. Contrast sensitivity loss is unavoidable in diffractive multifocal IOLs due to theoretical considerations; however, we consider this issue to be clinically insignificant. Our contrast sensitivity results, both with and without glare, indicate that the multifocal group was not inferior to the typical 60-year-old normal Japanese subjects evaluated by Takahashi whose contrast sensitivity was measured with the CGT1000 [29]. The contrast sensitivity of the multifocal group was at an acceptable level.

Approximately 85% of multifocal patients were spectacle-independent in our study, a rate consistent with the findings of previous reports of Tecnis® multifocal IOLs in which the percentage ranges from 82.6% to 92.8% [6], [24], [28], [30]. In contrast, the rate of spectacle independence in the monofocal group was approximately 12%. Intermediate and near spectacle dependency was significantly lower in the multifocal group, and distance spectacle dependency was also lower, although not significantly. Spectacle independence is the primary reason that patients choose to undergo multifocal IOL implantation; therefore, this result demonstrates that multifocal IOLs meet these expectations.

The NEI VFQ-25 measures the self-reported, vision-targeted health status of people with chronic eye diseases [31]–[33]. It has been used in normal subjects as well as those with ocular diseases, such as age-related macular degeneration, cataracts, glaucoma and Graves’ ophthalmopathy [32], [34], [35]. It has also been used to evaluate subjective visual function changes after various intraocular procedures, including cataract and macular hole surgery [36]. The Japanese version of the NEI VFQ-25 was validated by Suzukamo et al. [23]. In our study, the VFQ-25 scores exhibited no significant differences between the multifocal and monofocal groups, except for one instance in which the multifocal group rated poorer nighttime driving. These results suggest a limitation of the VFQ-25 test. Current cataract surgery is very sophisticated and results in a high level of satisfaction; therefore, it may be difficult to detect significant differences in patient satisfaction between the two types of implanted IOLs. However, our results can be interpreted from a different perspective: subjective visual performance of multifocal IOLs is not inferior to that of monofocal IOLs. On the other hand, the multifocal group scored significantly worse than the monofocal group in the dimension of nighttime driving. Patients with multifocal IOLs sometimes have difficulty in performing nighttime driving due to decreased contrast sensitivity, glare disability and/or the appearance of halos. Consequently, clinicians should be wary of implanting multifocal IOLs in patients who drive at night. Alio et al. reported that the VFQ-25 scores for near vision are significantly better in multifocal IOL-implanted patients than in monofocal IOL-implanted patients and that there are no significant differences in scores for nighttime driving between the two groups [25]. Their results, in contrast to ours, demonstrated superiority in terms of VFQ-25 scores in multifocal IOL patients. A probable reason for this discrepancy is national differences in attitudes towards eyewear. All of the subjects in our study were Japanese patients. In general, Japanese persons, especially those who select the implantation of monofocal IOLs, have less psychological resistance against glasses. They do not feel inconvenienced if they must read books or newspapers with glasses. We consider this phenomenon to explain why the near vision VFQ-25 scores of the monofocal group were not inferior to those of the multifocal group, although the mean UNVA was worse and the rate of spectacle dependency was higher in the monofocal group.

Multifocal IOLs have a different design concept than monofocal IOLs. The purpose of monofocal IOLs is to see the focal distance as clearly as possible; these lenses attempt to achieve high resolution and low aberration. On the other hand, multifocal IOLs are used to see distant and near objects without using spectacles; these lenses do not always seek to optimize resolution. Consequently, multifocal IOLs are inferior to monofocal IOLs in some respects. The VFQ-25 results of the present study demonstrate that the subjective visual performance of multifocal IOLs in implanted eyes is not inferior to that of monofocal IOLs; therefore, we do not consider any subjective disadvantages associated with using multifocal IOLs to be large, with the exception of nighttime driving. On the other hand, UNVA was much better and the rate of spectacle dependency was substantially lower in the multifocal group.

In conclusion, we compared the visual performance of multifocal IOLs and monofocal IOLs composed of the same optic materials and design. The mean UNVA was significantly better and the rate of spectacle dependency was significantly lower in the multifocal group. However, CIVA, CNVA and contrast sensitivity were better in the monofocal group. The VFQ-25 scores exhibited no significant differences between the two groups, with the exception that nighttime driving was scored as less problematic in the monofocal group. Aside from this exception, our results demonstrate that the multifocal IOLs used in this study decreased the spectacle dependency of patients without compromising the subjective visual function.
